# Human papillomavirus, gene mutation and estrogen and progesterone receptors in breast cancer: a cross-sectional study

**DOI:** 10.11604/pamj.2021.38.43.22013

**Published:** 2021-01-15

**Authors:** Abdallah Mohammed Elagali, Ahmed Abdelbadie Suliman, Mohammed Altayeb, Anas Ibrahim Dannoun, Narasimha Reddy Parine, Hader Ibrahim Sakr, Howayda Saeed Suliman, Moustafa Elsaeid Motawee

**Affiliations:** 1 Faculty of Medicine, Batterjee Medical College of Science and Technology, Jeddah, KSA,; 2 Histopathology and Cytology Department, Faculty of Graduate Study and Scientific Research, National Ribat University, Khartoum, Sudan,; 3 Pathology Department, Faculty of Medicine, Taibah University, Almadinah Almonawarah, Saudi Arabia,; 4 Molecular Genetics Department, Faculty of Medicine, Umm Al-Qura University, Mecca, KSA,; 5 Medical Genetics Department, Genome Research Chair, Department of Biochemistry, College of Science, King Saud University, Riyadh, KSA,; 6 Physiology Department, Kasr Al-Ainy Faculty of Medicine, Cairo University, Cairo Governorate, Egypt,; 7 Department of Biochemistry, Faculty of Medicine, Alexandria University, Alexandria, Egypt,; 8 Department of Histology and Cytology, Faculty of Medicine, Al-Azhar University, Cairo, Egypt

**Keywords:** Breast cancer, human papillomavirus, invasive lobular carcinoma, p53 gene, retinoblastoma gene, estrogen receptors, progesterone receptors

## Abstract

**Introduction:**

recent studies show a good relationship between breast cancer (BC) and human papillomaviruses (HPV) wich is responsible for about 18% of BC cases. This study aimed to assess the relationship between different genotypes of HPV and the expression of P53 and retinoblastoma (RB) genes and estrogen and progesterone receptors in BC among Sudanese women.

**Methods:**

one hundred and fifty tissue blocks were obtained from females diagnosed with BC. Positive samples were used to determine genotypes with an applied biosystem (ABI 3730XL) genetic analyzer for sequencing and immunohistochemistry.

**Results:**

13/150 samples showed HPV DNA. High-risk HPV-16 was detected in 5 cases, high-risk-HPV-58 was found in four cases, and HPV-18 was detected in three cases. Low-risk-HPV-11 was detected in a single invasive lobular carcinoma (ILC) case. P53 and RB gene mutations were detected in 35 and 30 BC cases, respectively. P53 gene mutation was frequently identified in grade (III) BC while RB gene mutation was positive in grade (II). Grade (II) BC had a higher incidence of HPV-16 and 58. On the other hand, HPV-18 had a higher incidence in grade (III). Estrogen and progesterone receptors were expressed in 94 and 79 HPV cases among the study group, respectively.

**Conclusion:**

this study elucidates the associations between HPV genotypes and BC. A statistically significant association was observed among p53 and RB gene mutations and different BC histological types. On the other hand, there was a statistically insignificant association between HPV genotyping and different BC gradings, BC histological types, P53 and RB genes mutations, and estrogen and progesterone receptor expression. Also, there was a statistically insignificant association among estrogen and progesterone receptors expression and BC grading. RB gene mutation was significantly associated with different BC grades. On the other hand, there was a statistically insignificant association between progesterone receptor expression and BC.

## Introduction

Breast cancer (BC) is the most common cancer in women and is the number one cause of cancer mortality in women worldwide. In 2008, about 1.38 million new BC cases were diagnosed in lower-income countries, representing almost half of all BC cases and nearly 60% of deaths [1]. BC's survival rates are widely variable, with an estimated 5-year survival of 80% in high-income countries to below 40% for low-income countries [2]. According to Globocan, about two million new cases were diagnosed in the United States in 2018 with expected deaths of 626,679 [3]. BC incidence increases in North Sudan tribes, with 16.3% in Galyean, 6.6% in Shygea, and 5.3% in Danagala [4]. Women living in rural areas in Sudan constituted 58% of individuals presented with BC than those in urban areas [5]. Many risk factors are associated with BC, mostly related to lifestyle and environmental considerations. Other factors include gender, age, genetic factors, dense breast tissue, and positive family history [6]. In Africa, women under the age of forty are most likely to succumb to BC [7]. The chance of a BC diagnosis after menopause is higher in women who are overweight or obese. Also, the use of diethylstilbestrol may increase the incidence of BC [8]. Early diagnosis is essential for effective treatment. Self-examination is the best technique for detecting breast inflammation or nodules and explaining sickness signs [9]. Radiologic studies, such as computed tomography, magnetic resonance imaging, or positron emission tomography, are crucial for preliminary diagnosis [10]. Breast tissue biopsy and microscopic examination can confirm the diagnosis. Fine needle aspiration cytology facilitates and accelerates clinical and histopathological recognition of BC [11]. Many techniques can also help diagnose, such as *in situ* hybridization [12] and immunohistochemistry [13].

Some viruses, such as Epstein-Barr virus, mouse mammary tumor virus, and human papillomavirus (HPV), have a role in BC development and progression [14]. HPV is linked to other types of cancers, such as head and neck, cervical, vulvar, vaginal, anal, and penile carcinomas [15]. The association between HPV and BC is not yet well established. The prevalence of HPV in BC ranges from 0 to 86% [16]. Several studies support HPV involvement in BC, but other studies could not detect HPV subtypes in BC tissues [17]. Clarification of the roles and mechanisms of HPV involvement in BC is needed. HPV is a small, circular, double-stranded DNA virus. Approximately 200 different HPV strains have now been identified and classified into mucosal and cutaneous forms. The mucosal HPV can be classified as low-risk (Lr) and high-risk (Hr) based on the propensity for malignant progression of lesions [18]. LrHPV subtypes include HPV-6 and HPV-11. These subtypes cause more than 90% of genital warts. HrHPV subtypes (e.g., 16, 18, and 58) cause squamous intra-epithelial lesions that can progress to invasive squamous cell carcinoma [17]. Contaminated hands can transmit HPV from the female perineum to the breasts [19]. HPV exhibits three oncogenes, E5, E6, and E7; two regulatory proteins, E1 and E2; and two structural proteins, L1 and L2 [20]. HPV E6 and E7 proteins are known for blocking pRb and p53 tumor suppressor genes, thus contributing to oncogenesis [21]. E6 binds to and degrades p53. E7 binds to and causes dysfunction of pRb. Significantly, hrHPV is integrated early into the host genome, and the structure of genes is identified. P53 possesses an N-terminal transactivation domain (TAD), a proline-rich region (PRR), a central DNA binding domain (p53C), a tetramerization domain (TET), and an extreme C terminus. The DNA binding domain is the location of most cancer-associated p53 mutations. Several human tumors show mutations and deletions of the RB gene (13q14), with inherited allelic loss of RB conferring increased predisposition to cancer formation [22]. Our study explored the association between HrHPV, p53, and RB gene mutation in BC among Sudanese patients. The prevalence of HrHPV was assessed using PCR, genotyping, and sequencing. Expression of p53 and RB gene mutations used immunohistochemical methods to examine correlations between HPV genotypes and the expression of mutant p53 and RB gene and tumor grades.

## Methods

**Study design**: this cross-sectional study design initially recruited 2000 patients with breast tumors. One thousand patients declined, 500 did not meet study criteria, sampling was insufficient for 100 patients, 50 were benign, and the remaining 150 participants were successfully included in the study after pathologist approval.

**Sampling method and recruitment**: formalin-fixed paraffin wax processed blocks were investigated. The sample collection technique was based on a randomization formula. N = (2 x Joint Success rate x Joint Failure rate) x (Z_Þ_+ Z_ß_)^2^/(Diff)^2^

**Study site**: the samples were collected from histopathology laboratories at the Radiation Isotope Center, Bahri and Al-Ribat Hospital, Khartoum State, Sudan. This facility is the central referral system for the diagnosis and treatment of patients with breast tumors.

**Data collection**: the study was conducted between January 2015 and December 2016. The average monthly rate of newly diagnosed cases is about 90%. Interpretation of BC grading was reported, according to the Bloom-Richardson (Nottingham) Grading System: 1) Grade 1 or well-differentiated cells (scores 3, 4, or 5): grow slower and look more like normal breast tissue. 2) Grade 2 or moderately differentiated cells (score 6, 7): between Grades 1 and 3. 3) Grade 3 or poorly differentiated cells (score 8, 9): grow rapidly and look very different from normal cells [23]. Data were collected for age, histological type and tumor type (malignant or benign), and grade. Master sheets were used to record all PCR and immunohistochemistry results.

### Laboratory analysis

**Immunohistochemistry (IHC)**: followed the method of Pu *et al*. [24]. Paraffin-embedded blocks of breast tissues were retrieved from histopathology laboratories. The sections were mounted on poly-L-lysine-coated slides and dried in a hot air oven at 60°C for 1 hour. Sections were dewaxed in xylene and rehydrated in descending grades of ethyl alcohol. Sections were then washed three times with 1% phosphate buffer saline (PBS). Sections were boiled in Target Retrieval Solution of Dako (Real Envision Detection Kit) in a water bath at 95°c for 30 min. Sections were then blocked with 3% hydrogen peroxide in the dark. The following antibodies (Abs) were used: 50-100 microliters of primary mouse monoclonal mutant (RB Ab-1) antibody (Catalog no MS-107-B) or primary mouse monoclonal mutant (p53 Ab-8) antibody (Catalog no MS-738-P0, Thermo Scientific Company) at working dilutions of 1/100 for 30 min at room temperature; 50: 100 microliters of a secondary antibody (Thermo Scientific Company) were added. Immune reactivity was detected using diaminobenzidine (DAB) (Thermo scientific Company); 50: 100 microliters of DAB working solution was added and incubated at room temperature for 10 minutes. Finally, sections were counterstained with Mayer's Hematoxylin, dehydrated and cleared in xylene, and mounted with DPX. For each IHC assay, proof slides were used for negative and positive control. IHC stained sections were examined under light microscopy (Olympus CHT, Optical. Co.Ltd, Japan) using 4×, 10×, 40× 100×, objective and eyepieces of 10×, giving a maximum magnification of 1000.

**Mutated P53 and RB** were observed only as nuclear staining of epithelial cells, and nuclei with clear brown color were scored as positive. Two investigators scored the intensity of IHC staining for each marker based on subjective evaluation of color exhibited by antigen, antibody, and chromogen complex. Scores were 0 for negative (no color), 1+ for weak (light brown color), 2+ for moderate (dark brown color), 3+ for strong staining (dark brown color), and 4+ for overexpressing (very strong dark brown color). Scores of 0 or 1 were defined as negative, 2, 3 or 4 as positive.

**DNA extraction** was performed using a DNA FFPE Tissue Kit [25].

**Figure 1:**
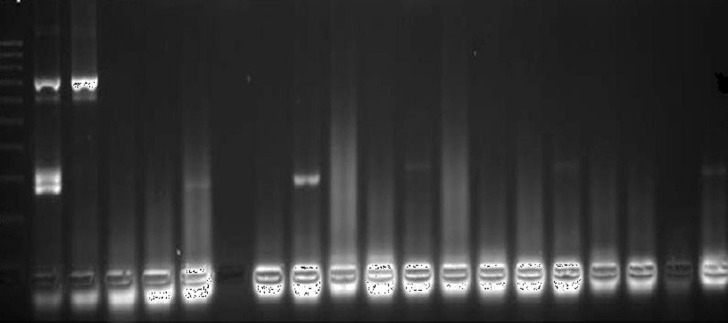
PCR amplification of HPV in breast cancer samples with a gel documentation system

**Amplification** (Table 1 and Figure 1) and interpretation (Table 2) used the HPV kit from Sacace Technologies [26]. The length of specific amplified DNA fragments was HPV- 267-325 bp.

**Table 1:** PCR results for controls from the HPV kit

Control	Step of the test controlled	Specific bands In the gel 267-325 bp	Specific bands In the gel 723 bp	Interpretation
**Neg. Control**	DNA isolation	No	No	Valid result
**DNA- buffer**	Amplification	No	No	Valid result
**Internal Control**	Amplification	No	Yes	Valid result
**HPV C+**	Amplification	Yes	No	Valid result

**Table 2:** different HPV Genotype sequences (5´-3´) detected in BC cases using an ABI 3730xL DNA analyzer (Applied Biosystems, Foster City, CA, USA)

HPV Genotype	Sequence (5´-3´)
**16**	**TTTTGTTACTGTGGTAGATACTACACGCAGTACAAATATGTCATTATGTGC TGCCATATGTACTTCAGAACCTACATATAAAAATACTAACTTTAAAGAGTA CCTACGACAAGGGGAGGAATATGATTTACAGTTTATTTTTCA**
**18**	**TTTTGTTACTGTGGTAGATACTACTCGCAGTACCAACTTAACAATATGTGC TTCTACACAGTCTCCTGTACCTGGGCGATATGATGCTACCAAATTTAAGCA GTATAGCAGACATGTTGAGGAATATGATTTACAGTTTATTTT**
**58**	**ATTTGTTACCGTAGTTGATACCACTCGTAGCACTAATATGACATTATGCAC TGAAGTAACTAAGGATGGTACATATAAAAATGATAATTTTAAGGAATATGT ACGTCATGTTGAAGAATATGACTTACAGTTTGTTTTTCA**
**11**	**TTGTTACTGTGGTAGATACCACACGCAGTACAAATATGACACTATGTGCAA CTGTGTCTAAATCTGCTACATACACTAATTCAGATTATAAGGAATACATGC GCCATGTGGAGGAGTTTGATTTACAGTTTATTTTTCA**

**Statistical analyses**: data were coded and entered using the statistical package for the Social Sciences version 25 (IBM Corp., Armonk, NY, USA). Data were summarized using frequency and relative frequency for categorical data. For comparing categorical data, Chi-square (2) tests were used. Exact tests were used instead when the expected frequency was less than 5 [27]. P-values less than 0.05 were considered statistically significant.

**Ethical consideration**: the institutional research board of Ribat University, Sudan, approved this study on May 9, 2015. Before data collection, study objectives were discussed thoroughly with the local authorities and staff. Data were collected anonymously, and confidentiality was guaranteed. The study protocol was compliant with the declaration of Helsinki.

## Results

A total of 150 BC samples were analyzed for the presence of HPV DNA with PCR. All negative specimens were considered as baseline characteristics. Cases were further subdivided into medullary carcinoma, invasive lobular carcinoma (ILC), mucinous carcinoma, and invasive ductal carcinoma (IDC). The association of HPV DNA with BC cases was statistically insignificant.

**Figure 2:**
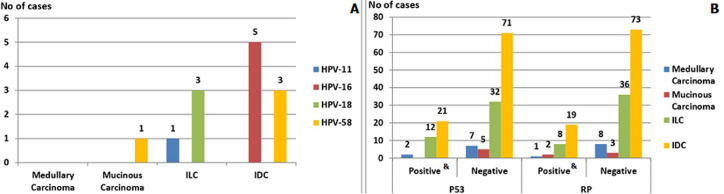
association between different histological types of BC and (A) Human papilloma virus (HPV) genotypes and (B) P53 and RB gene mutations (IVC: invasive lobular carcinoma, RB: retinoblastoma); (&): statistically significant (p < 0.05)

**Association between HPV genotypes and different histological types of BC among the study groups**: the association of HPV-DNA with BC cases was statistically insignificant (p = 0.7308) in the form of thirteen HPV-positive cases out of one hundred and fifty BC cases. We can notice from (Figure 2A) that HPV-positive cases were distributed as follows: 1) five high-risk HPV-16-positive cases in the form of IDC. 2) Four high-risk HPV-58-positive cases in the form of one mucinous Carcinoma and three IDC. 3) Three high-risk HPV-18-positive cases in the form of ILC. 4) Only one low-risk HPV-11-positive case in the form of ILC.

**Association among P53 and RB genes mutations and different histological types of BC among the study groups**: P53 gene mutation was detected in thirty-five patients, twelve ILC, twenty-one IDC, and two medullary carcinoma cases. No P53 gene mutations were found in mucinous carcinoma tissues. On the other hand, RB gene mutation was detected in thirty patients, nineteen IDC, eight ILC, two mucinous carcinomas, and a single medullary carcinoma case. This reflects a statistically significant association between P53 (p = 0.0055*) and RB (p = 0.0154*) genes mutations and BC histological types (Figure 2B).

**Figure 3:**
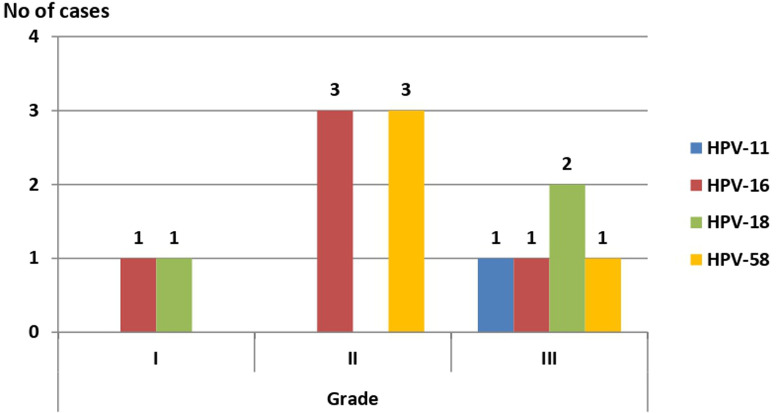
association between different HPV genotypes and BC grading in the study groups

**Associations among HPV genotypes and BC grading among the study groups**: two, six, and five HPV positive cases in grades (I), (II), and (III) were found among 150 BC tissues, respectively. A higher incidence of HPV-16 was found in grade (II) (three cases) than in grades (I) and (III) (a single case in each). HPV-58 showed a similar profile with three positive cases in grade (II) and a single case in grade (III). On the other hand, HPV-18 showed a higher incidence in grade (III), with two positive cases, than in grade (I), with a single positive case, while no positive cases were found in grade (II). HPV-11 was positive only in a single grade (III) case. The association between different HPV genotypes and different BC grades was statistically insignificant (Figure 3, p = 0.580).

**Figure 4:**
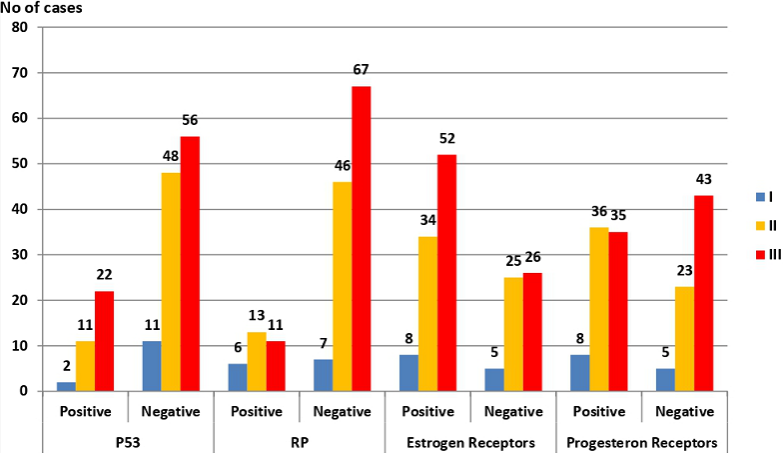
association among P53 and RB gene mutations, estrogen, progesterone receptor expression and BC grading (RB: retinoblastoma)

**Figure 5:**
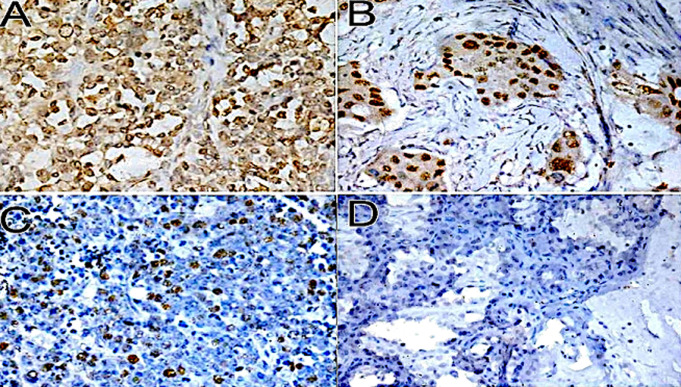
p53 gene mutation immunohisto chemistry staining of BC tissue (40 X) showing; (A) strong positive (4+), (B) moderate positive (3+), (C) weak positive (2+), and (D) negative (1+)

**Figure 6:**
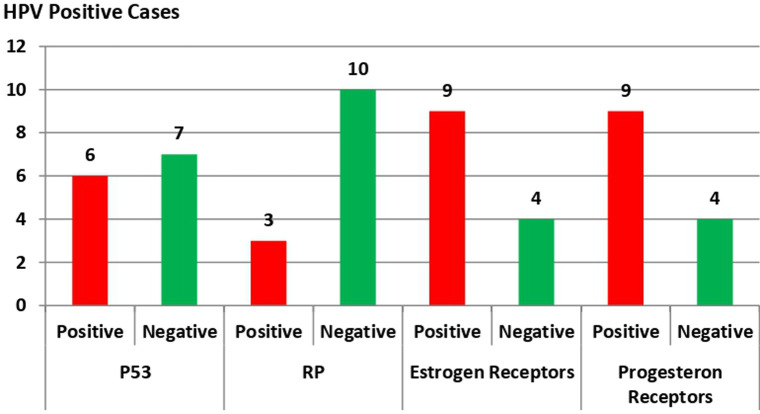
RB gene mutation immunohisto chemistry staining of BC (40 X) tissue showing; (A) strong positive (4+), (B) moderate positive (3+), (C) weak positive (2+), and (D) negative (1+)

**Associations among P53 and RB genes mutations and BC grading among the study groups**: P53 gene mutation was positive in thirty-five BC cases, distributed as two grade (I), eleven grade (II), and twenty-two grade (III) cases. The association between P53 gene mutation and BC cases appeared to be statistically insignificant (p = 0.144). RB gene mutation was positive in a total of thirty BC cases and was distributed as six grade (I) cases, thirteen grade (II) cases, and eleven grade (III) cases. The association between BC grading and RB genes mutation appeared to be statistically insignificant (p = 0.0599) (Figure 4, Figure 5, Figure 6).

**Associations among estrogen and progesterone receptor expression and BC grading among the study groups**: estrogen receptors were expressed in a total of ninety-four BC cases, including eight grade (I), thirty-four grade (II), and fifty-two grade (III) cases. Such expression appeared to be statistically insignificant (p = 0.449). Progesterone receptors were expressed in a total of seventy-nine BC cases, including eight grade (I) cases, thirty-six grade (II) cases, and thirty-five grade (III) cases. The association between progesterone receptor expression and BC grade was also statistically insignificant (p-value = 0.247) (Figure 4).

**Figure 7:**
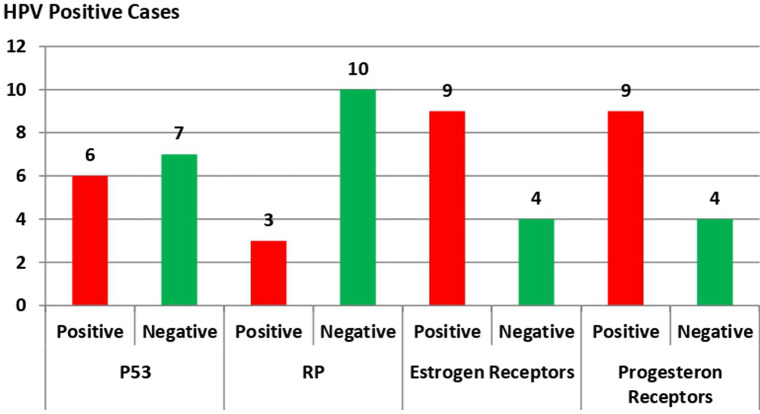
association between P53 and RB genes mutations, estrogen, and progesterone receptor expression and HPV positive cases among the study groups Human Papilloma Virus (HPV), base paring (bp), Invasive Ductal Carcinoma (IDC), Invasive Lobular Carcinoma (ILC), Retinoblastoma (RB), strong positive (A), moderate positive (B), weak positive (C), negative (D), Statistically significant (p < 0.05)

**Associations among P53 and RB genes mutations, estrogen and progesterone receptor expression, and HPV positive cases among the study groups**: P53 and RB gene mutations were detected in six and three HPV positive BC cases, respectively, which was statistically insignificant with p-value = 0.089 for P53 and 0.568 for RB gene mutations. Also, both estrogen and progesterone receptors were positively expressed in nine HPV positive BC cases and statistically insignificant correlation (p = 0.522 and p = 0.129, respectively) (Figure 7).

## Discussion

BC is a serious health problem worldwide. Viral pathogens are extensively explored for BC, but a moderate association between HPV and BC suggests a potential to improve early diagnosis, prevention, treatment, and follow-up. Many studies have investigated HPV genotypes in BC in different parts of the world, but no such studies are available for Sudan. This study examined the association of variables HPV integrations, P53 and RB gene mutations, and estrogen and progesterone receptor (ER/PR) expression with BC among Sudanese women. In the current study, a statistically insignificant association was observed between HPV infection and women diagnosed with BC; with an 8.67% incidence of HPV. Previous studies showed 0 to 86% HPV prevalence in BC worldwide [28,29]. Similar studies succeeded in detecting HPV in BC in Thailand [30] and China [14]. Conversely, studies in Spain [31] and India [32] failed to detect HPV in BC due to several factors related to the used techniques or a different genetic population. Our study provides additional evidence that HPV may play a role in the development of BC [33]. Our results showed an HPV incidence of 8.69% for IDC, which is much lower than 21%, 29%, and 35.41% incidences in the studies conducted by Heng *et al*. [28], Salman *et al*. [34], and Francis *et al*. [35], respectively. HPV was also found in 14% of ILC cases in the Salman *et al*. study [34] compared to 9.09% in our study. This higher incidence is affected by the number of samples among UK patients and fresh samples.

HPV-16 is the most common genotype in breast tumors [30]. Our study showed a statistically insignificant association between HPV-16 and the five cases of IDC-BC with an incidence of 3.33%. A study conducted in Iraq in 2017 found a 25.6% incidence of HPV-16 in BC [36]. Lawson *et al*. conducted a study on twenty cases of BC found a 10% incidence of HPV-16 [37]. The present study showed no significant association among BC samples and the three ILC cases exhibiting HPV-18. Al-Awany *et al*. found a 27.1% association of HPV-18 and BC [38]. Our study shows no statistically significant association (2.67%) with HPV-58. A study conducted by Ling *et al*. showed a higher incidence [39]. Our study found no statistically significant association (0.67%) with HPV-11, in the form of a single ILC case. In Kuwait, the study delivered by Francis *et al*. detected a 13.7% association of HPV-11 in IDC [35]. Regarding IDC, this study found that HPV-16 and HPV-58 had the highest incidence of 5.43% and 3.26%, respectively. Hong and Tang reported a 51.1% incidence of HPV-16 [40]. In another study by Wang *et al*. the incidence of HPV-58 was 35.6% [41]. Moreover, the current study demonstrated that HPV-18 and HPV-11 were presented more in ILC with percentages of 6.82% and 2.27%, respectively. In Syrian women, HPV-18 was found in 37.16% of ILC cases [40]. However, Wang *et al*. found HPV-18 DNA more often in IDC than ILC, with a prevalence of 46% [41]. A third study in Isfahan showed that HPV-11 was the most common LrHPV in IDC and not ILC, with a prevalence of 3.6% [42].

A statistically significant association was observed among p53 and retinoblastoma (RB) gene mutation, and different BC histological types, with percentages of 23.33% and 20%, respectively. Bertheau and colleagues found that p53 gene mutations are the most frequent genetic alterations in and different BC histological types, with an incidence of 30% [43]. Conversely, Masri *et al*. did not find any association between p53 gene mutation and BC samples obtained from 20 BC patients [44]. Our study's findings are consistent with Anderson *et al*. who found the RB gene mutation in 21% of cases [45]. The present study indicates that p53 gene mutation was 27.3%, 22.8%, and 20.7% for ILC, DLC, and medullary carcinoma, respectively. RB gene mutation incidences were 40%, 20.7%, 18.22%, and 11.1% for mucinous carcinoma, IDC, ILC, and medullary carcinoma, respectively. A study performed by Hong and Tang [40] found the expression of p53 gene mutation 46.7% in IDC. Our findings for RB gene mutation are consistent with an Italian study performed in 2009 [46]. No statistically significant association was identified between HPV genotypes and BC grading. HPV-16 had a higher incidence in grade I (7.69%), HPV-58 had a higher incidence in grade II (5.08%), HPV-18 showed relatively high incidence in grade I (7.69%), and HPV-11 was found only in grade III cases (1.28%).

P53 gene mutation was not significantly related to different BC grades, with twenty-two positive cases in grade (III), eleven cases in grade (II), and two cases in grade (I), out of thirty-five P35 gene mutation-positive cases. This finding is slightly different from a study conducted by von Deimling *et al*. who observed p53 gene mutation in eleven cases out of twenty-two grade (II) and (III) BC cases [47]. Also, the RB gene mutation was statistically insignificantly associated with different BC grades. ER and PR were not statistically significantly associated with BC grades. ER incidence was 61.54%, 57.63%, and 66.67% for BC grades II, III, and I, respectively, in ninety-four positive cases. The PR incidence was 38.46%, 42.37%, and 33.34% for BC grades II, III, and I, respectively, in seventy-nine positive cases. The role of ER/PR in deciding the management and assessing the prognosis of BC is well-established [48]. Our study also found that P53 and RB gene mutations were positive in six and three out of thirteen HPV positive BC cases, respectively. Both ER/PR were positive in nine out of thirteen HPV positive BC cases. Two studies by Hong and Tang [40] and Wang *et al*. [41] reported statistically significant expression of HPV-DNA associated with expression of p53 gene mutations. The primary transforming capacities of HPV originate from the E6 and E7 proteins. The tumor suppressor p53 initiates checkpoints causing cell cycle arrest or induces apoptosis. HPV E6 oncoprotein initiates degradation of p53 [49]. Direct binding of E7 to pRB impairs its function [50]. Moreover, the E7 can impair p53 function even in the absence of E6 [51].

## Conclusion

A statistically insignificant association among HPV, both generally and for different genotypes, and estrogen and progesterone receptors was observed for different histological types and grades of BC among the study group of Sudanese women. A statistically significant association among p53 and RB genes mutation with different histological types of BC was found. Further studies with larger sample sizes are recommended to measure the real burden of HPV in BC etiology in Sudan.

### What is known about this topic

- Breast cancer is the most common cancer among Sudanese women;
- HPV is the most common in Sudan linked to other types of cancers;
- Gene mutation, one of the causal agents of cancer.

### What this study adds

- There is a high-risk HPV 16, 18, 58, and low-risk HPV11 sequencing with breast cancer and grades in Sudan;
- We identified in high-risk populations, p53 and RB gene mutations can be used to screen breast cancer is particularly noteworthy and have important implications for screening programs in Sudan.
